# Association study of rs1801282 *PPARG* gene polymorphism and immune cells and cytokine levels in a Spanish pregnant women cohort and their offspring

**DOI:** 10.1186/s12929-020-00694-3

**Published:** 2020-11-30

**Authors:** Maria García-Ricobaraza, Mercedes García-Bermúdez, Francisco J. Torres-Espinola, M. Teresa Segura Moreno, Mathieu N. Bleyere, Ligia E. Díaz-Prieto, Esther Nova, Ascensión Marcos, Cristina Campoy

**Affiliations:** 1 Department of Paediatrics, School of Medicine, Universidad de Granada, Granada, Spain; 2 Instituto de Investigación Biosanitaria ibsGRANADA, Health Sciences Technological Park, Granada, Spain; 3 EURISTIKOS Excellence Centre for Paediatric Research, Universidad de Granada, Granada, Spain; 4 Department of Physiology, Haematology and Immunology, Nangui Abrogoua University, Abidjan, Côte d’Ivoire; 5 Institute of Food Science, Technology and Nutrition (ICTAN), CSIC, Madrid, Spain

**Keywords:** Pro12Ala *PPARG* polymorphism, Immune system, Pregnant mothers, Newborn cord blood

## Abstract

**Background:**

Peroxisome proliferator activated receptor gamma (*PPARG*) belongs to the nuclear receptor superfamily functioning as transcription factors to regulate cellular differentiation, development and metabolism. Moreover, it has been implicated in the regulation of lipid metabolism, as well as the maturation of monocytes/macrophages and the control of inflammatory reactions. The aim of this study was to evaluate the relationship between the Pro12Ala (rs1808212) *PPARG* gene polymorphism on immune molecular and cellular components in mothers and their offspring participating in the PREOBE study.

**Methods:**

DNA from maternal venous blood samples at 24, 34 and 40 gestational weeks, plus cord blood samples was extracted. Pro12Ala *PPARG* polymorphism genotyping was performed, and immune system markers were analyzed by flow cytometry.

**Results:**

Study findings revealed no effect of rs1808212 *PPARG* genotypes on innate immune parameters in mothers and their offspring; however, CD4 + /CD8 + ratio were decreased at 24 and 34 weeks in pregnant women carrying the *CG* (Pro12Ala) rs1808212 polymorphism, (p = 0,012 and p = 0,030; respectively). Only CD19 levels in peripheral blood were significantly higher at delivery in pregnant women carrying the *CC* (Pro12Pro) genotype (p ≤ 0.001). Moreover**,** there were statistically significant differences in leukocytes and neutrophils maternal levels at 34 weeks of gestation, being lower in carriers of Pro12Ala genotype (p = 0.028 and p = 0.031, respectively).

**Conclusions:**

Results suggest that Pro12Ala *PPARG* polymorphism may have an effect on some cell and immune parameters in pregnant women during pregnancy and at time of delivery. However, newborn innate immune system does not seems to be influenced by *PPARG* Pro12Ala polymorphism in cord blood.

## Background

The immune system is our first line of defense against microbial infection. It is composed of leukocytes that secrete various immune molecules such as cytokines, chemokines, immunoglobulins, and complement proteins in order to eliminate pathogens as well as mediate cell-to-cell communication. A successful pregnancy requires a robust, dynamic and responsive immune system. Immune modulation and tolerance induction are required at the beginning of the pregnancy to allow implantation, but a successful pregnancy also depends on a responsive immune system that can protect both the mother and the fetus against environmental insults when necessary [1]. Indeed, for a successful pregnancy outcome, the maternal immune system should have reinforced networks that can recognize danger signals, appropriately eliminate them and promote repair if needed. In addition to the maternal immune system, the fetal–placental unit has an actively developing immune system that can further modify the maternal immune response and how the maternal immune system reacts to the environment. As such, the immunological milieu during pregnancy is unique, modulated and dynamic, and definitely not suppressed [2].

Peroxisome proliferator activated receptors (*PPAR*s) family belong to the nuclear receptor superfamily that functions as transcription factors to regulate cellular differentiation, development and various metabolic processes [3], especially lipid and glucose homeostasis [4]. To date, three *PPAR*s have been identified: alpha (*PPARA*), delta (*PPARD*) and gamma (*PPARG*). *PPARG* is highly expressed by adipocytes, skeletal muscles, liver and kidney, and to a lesser extent in colon and the immune cells–macrophages. *PPARG* gene is located on chromosome 3, contains 9 exons and expands more than 100 kb. A missense mutation resulting in an alanine substitution for proline at codon 12 (Pro12Ala) of the *PPARG* gene (rs1801282) is located close to the NH_2_-terminal region of the protein, in the ligand independent activation domain, and has been found to modulate *PPARG* transcriptional activity. A reduced transcriptional activity of the *PPARG* gene as a result of Pro to Ala aminoacid substitution has been demonstrated [5]. Multiple diverse mechanisms converge on the activation function domain to regulate the transcriptional activity, and insulin sensitizing potential, of *PPARG* [6]. Activation of PPAR signaling pathways lead to an effect in lipid synthesis and oxidation, glucose uptake, inflammation, and expression of immunoregulatory genes. PPARG has a role in the maintenance of regulatory T cell numbers [7], as well as maturation of monocytes/macrophages and control of inflammatory reactions [3, 8, 9]. Differentiation and activation of these immune cells are linked to this nuclear hormone receptor [10, 11]. *PPARG* is expressed in various immune system cell types; both the innate and the adaptive immune system are strongly influenced by PPAR activation triggering macrophages and other leukocyte populations, such as lymphocytes and dendritic cells [12].

Obesity is a metabolic disease that results in a chronic low-grade inflammation status, characterized by high levels of inflammatory mediators, such as C-reactive protein, interleukin (IL) 6, and TNF [13]. Once considered a high-income country problem, overweight and obesity are now on the rise in low- and middle-income countries, particularly in urban settings [14]. Excess weight/adiposity is associated with several co-morbidities [15]. High rates of obesity in women of childbearing age [16, 17] has made obesity the most common medical problem in pregnancy. A high body mass index (BMI) entering pregnancy and excess weight gain during pregnancy can exacerbate the natural inflammatory state associated with pregnancy resulting in detrimental health outcomes for the mother [18, 19], such as gestational diabetes, pre-eclampsia and delivery of macrosomic infants [20]. Furthermore, children born to women who enter pregnancy in an obesogenic state are at higher risk for several adverse long-term health outcomes in comparison to children born to lean mothers [21]; maternal obesity is associated with increased incidence of cardiovascular disease, asthma, and diabetes in the offspring, thus, maternal obesity likely disrupts normal development and maturation of the offspring’s immune system in utero [13]. Obesity is a multifactorial condition, influenced by genes, lifestyle and environmental factors. Approximately 40–70% of variation in obesity is attributed to genetic factors [22, 23]. While much of this genetic etiology remains unidentified, numerous studies have identified loci that are associated with in the general population. SNPs in *FTO* gene have been found significantly associated with pre-pregnancy obesity and body mass index in pregnant women [24]. Moreover, relationship between gene polymorphisms in *PPARG* [25] and overweight and obesity have also been reported. Recently, a direct effect on the weight gain during pregnancy has been reported associated to modified transcriptional activity of *PPARG* gene [26].

The main objective of this study is to examine the possible influence of *PPARG* Pro12Ala (rs1808212) polymorphism in pregnant women and their offspring on some immunological and cellular parameters during pregnancy and at birth.

## Materials and methods

### Study design and participants

Subjects participated in a Spanish prospective, observational, longitudinal study: “Study of Maternal Nutrition and Genetics on the Foetal Adiposity Programming”, PREOBE study (retrospectively registered at ClinicalTrials.gov; Identifier: NCT01634464). Briefly, healthy pregnant women with singleton pregnancies and age between 18 and 45 were assessed for eligibility between 12 and 20 weeks’ gestation, as previously described [27, 28]; 145 pregnant women and their offspring were eligible for the present study, although only 137 mothers were genotyped and analyzed, due to non-Spanish background of some of them, or having more than 1 child enrolled in the study, thus avoiding bias. Maternal age, pre-conceptional BMI, presence of gestational diabetes mellitus (GDM), smoking and alcohol habits, gestational age at delivery, gestational weight gain (GWG), mode of delivery, parity, placental weight, neonatal anthropometry (birth weight, birth length, and head circumference) and newborn sex was recorded.

All experimental procedures were approved by the Ethics Committees of the Universidad de Granada, and Mother-Infant and San Cecilio University Hospitals of Granada, Spain, and performed in line with the Declaration of Helsinki II Principles. Written informed consent was obtained from all participant women prior to enrollment in the study.

### Pro12Ala (rs1808212) *PPARG* polymorphism genotyping.

Genomic DNA was obtained from blood leukocytes—at 24, 34 weeks and at delivery—and venous umbilical cord blood, using phenol chloroform extraction protocol, and quantified by 0.8% agarose gel electrophoresis. Genotyping was performed using TaqMan™ SNP Genotyping Assay C_1129864_10 (Applied Biosystems, Foster City, CA, USA) on an ABI PRISM 7900HT Sequence Detection System according to manufacturers' instruction kit and manual (Applied Biosystems, Foster City, CA, USA).

### Cells blood count-differential and lymphocyte phenotype

White blood cell (WBC) profiles were determined with an automatic haematology analyzer (ADVIA 2120 S Healthcare, Spain). For lymphocyte subsets analysis, blood aliquots were taken into 1.0 ml plastic tubes and diluted 1:1 with Cyto-Chex® (Streck Cell Preservative™ CE). Briefly, blood aliquots were incubated with monoclonal antibodies (BD Biosciences, San José, CA, USA), to differentially label CD45^+^ (the pan-leukocyte marker), CD3^+^ (T mature cells), CD4^+^ (T helper cells), CD8^+^ (T cytotoxic cells), CD19^+^ (B cells), and CD16^+^56^+^ (Natural Killer (NK) cells) for 30 min at room temperature. Red blood cells were lysed with lysing solution (Becton Dickinson), washed in phosphate buffered saline and resuspended. Lymphocytes were gated by forward and side scatter and pan-leukocyte marker expression (CD45) and by four-staining procedure (CD3-FITC/CD16 + 56-PE/CD45-PerCP/CD19-APC and CD3-FITC/CD8-PE/CD45-PerCP/CD4-APC) BD Pharmigen™ fluorochrome conjugated and analysed by flow cytometry (FACScan Plus Dual Laser, Becton Dickinson, Sunnyvale, CA, USA).

Serum samples were used for quantification of IL-6, IL-10, and TNF. A high sensitivity multiple analyte commercial kit (HSCYTO-60-SK, Millipore, USA) was used with xMAP technology from Luminex® Corporation. Minimum detectable concentration was 0.10 pg/mL for IL-6, 0.15 pg/mL for IL-10 and 0.05 pg/mL for TNF. Low and high concentration quality controls were ran in the assay.

### Statistical analysis

All statistical analyses were performed using SPSS v22.0 statistical software package for Windows (IBM SPSS Inc., Chicago, IL, USA). To assess the data for normality, a Shapiro–Wilk test was performed. Descriptive statistics were mean and standard deviation (SD) for normal variables, and percentages for categorical ones. Comparison between rs1801282 *PPARG* genotypes using U Mann–Whitney test for non-parametric distribution or Student T test assuming equal variances with Levene test, were performed. Statistical significance level was set at p < 0.05.

All genotype data were checked for deviation from Hardy–Weinberg equilibrium (HWE) using http://ihg.gsf.de/cgi-bin/hw/hwa1.pl.

## Results

General characteristics of pregnant mothers participating in the current study are presented in Table 1. There were no statistically significant differences (p > 0.05) in demographic characteristics or pregnancy parameters (gestational age, GWG, gestational diabetes development, placenta weight) and presence of Pro12Pro or Pro12Ala genotypes in mothers. As shown in Table 1, there were no maternal subjects presenting the GG genotype; allele frequencies were 93.07% C and 6.93% G, and rs1801282 genotype distribution was in HWE (p = 0.38). The Ala12 allele frequency in our population was comparable to previous studies in Spanish [29] and other Caucasian populations (5.9–21.6%) [30, 31].

**Table 1:** Maternal characteristics of study participants based on *PPARG* Pro12Ala (rs1801282) genotypes

Characteristics		Pro12Pro (*CC*) (n:118)	Pro12Ala (*CG*) (n:19)	*p*
Maternal age (years)		31.54 ± 4.94	31.58 ± 4.46	0.976
Pre-conceptional BMI (kg/m^2^)		26.93 ± 5.32	26.17 ± 5.39	0.565
Cultural level	Primary/secondary (%)	60.17	57.89	0.851
	University (%)	39.83	42.11	
Family civil status	Single (%)	4.24	0	0.361
	Married/living with partner (%)	95.76	100	
Diabetes (%)		30.51	26.32	0.711
Smoking during pregnancy (%)		15.79	11.11	0.607
Alcohol consumption during pregnancy (%)		5.22	5.56	0.952
Gestational age (week)		39.58 ± 1.23	39.68 ± 1.16	0.737
Weight gain during pregnancy (kg)		10.42 ± 7.20	9.01 ± 6.88	0.455
Mode of delivery	Eutocic (%)	57.29	76.47	0.195
	Dystocic (%)	17.70	17.65	
	C-section (%)	25	5.88	
Parity (%)	0	58.12	36.84	0.084
	≥ 1	41.88	63.16	
Placental weight		513.73 ± 126.77	482.35 ± 108.49	0.338
Newborn sex	Boy/girl (%)	47.86/52.14	63.16/36.84	0.216
Glucose (mg/dl)		92.59 ± 32.61	90.50 ± 26.74	0.809
HbA1c (%)		4.86 ± 0.52	4.65 ± 0.42	0.135
Total cholesterol (mg/dl)		245.76 ± 49.73	253.13 ± 44.10	0.579
Triglycerides (mg/dl)		223.17 ± 80.71	238.06 ± 54.72	0.480
HDL-cholesterol (mg/dl)		68.67 ± 18.33	72.38 ± 13.12	0.441
LDL-cholesterol (mg/dl)		134.19 ± 41.07	133.13 ± 34.53	0.922

Results are expressed as mean ± standard deviation, unless otherwise stated. Bold: P-value < 0.05. *HDL* high density lipoproteins, *LDL* low density lipoproteins

Considering babies born to mothers of the study, there were no statistically significant differences in length (50.49 ± 2.13 vs. 50.10 ± 2.66, p > 0.05) or cephalic perimeter (34.58 ± 1.43 vs. 34.50 ± 1.54, p > 0.05) between those who were born to mothers carrying *CC* or *CG* genotypes, respectively. Regarding newborn weight, babies whose mothers carried *CG* genotype were heavier than those born to mothers carrying *CC* genotype (3329.66 ± 422.12 *vs*. 3531.05 ± 481.25), although statistical significance was marginal (p = 0.061).

Concerning immunological parameters, comparison between Pro12Ala/*CG *vs. Pro12Pro/*CC* mothers’ genotype was done at different gestational weeks (Table 2). In general, data show an increase in levels of some of the immune cells analyzed, such as leukocytes, neutrophils and monocytes, and decrease in levels of others (eosinophils lymphocytes, and large unstained cells (LUC) percentage) along gestation. Furthermore, levels of lymphocyte cells antigen positive to cluster of differentiation 3, 4, 8 and 19 (CD3, CD4, CD8 and CD19) seem to be decreased along pregnancy. No changes in levels of basophils or NK cells have been noted in our study cohort. All determined biological parameters which were reported in our study, were normal values according to clinical reference values.

**Table 2:** Immunological parameters in mothers according to rs1808212 *PPARG* genotypes along pregnancy

Immunological parameters	24 Weeks		*p*	34 Weeks		*p*	40 Weeks		*p*
	Pro12Pro (*CC*) n = 87	Pro12Ala (*CG*) n = 15		Pro12Pro (*CC*) n = 115	Pro12Ala (*CG*) n = 19		Pro12Pro (*CC*) n = 91	Pro12Ala (*CG*) n = 16	
Leukocytes (10^3^/µl)	10.68 (10.11–11.24)	9.41 (7.95–10.87)	0.09	**10.36 (9.93–10.79)**	**9.12 (8.23–10.01)**	**0.028**	13.44 (12.51–14.36)	14.38 (12.13–16.63)	0.43
Neutrophils (10^3^/µl)	7.98 (7.50–8.46)	6.99 (5.78–8.20)	0.12	**7.68 (7.32–8.03)**	**6.65 (5.83–7.48)**	**0.031**	11.22 (10.32–12.11)	12.03 (9.85–14.22)	0.48
Monocytes (10^3^/µl)	0.50 (0.47–0.54)	0.48 (0.40–0.56)	0.63	0.55 (0.52–0.58)	0.49 (0.44–0.55)	0.18	0.54 (0.49–0.58)	0.55 (0.44–0.65)	0.85
Eosinophils (10^3^/µl)	0.20 (0.17–0.23)	0.18 (0.14–0.22)	0.66	0.16 (0.14–0.18)	0.16 (0.12–0.19)	0.97	0.11 (0.09–0.13)	0.13 (0.09–0.16)	0.60
Basophils (10^3^/µl)	0.04 (0.034–0.047)	0.05 (0.035–0.073)	0.12	0.044 (0.040–0.049)	0.053 (0.038–0.067)	0.13	0.044 (0.037–0.050)	0.055 (0.040–0.051)	0.16
Lymphocytes (10^3^/µl)	1.83 (1.72–1.93)	1.61 (1.34–1.89)	0.12	1.79 (1.70–1.88)	1.62 (1.42–1.83)	0.18	1.39 (1.25–1.53)	1.48 (1.13–1.82)	0.64
CD3 (10^3^/µl)	1.34 (1.26–1.42)	1.20 (0.98–1.42)	0.17	1.33 (1.27–1.40)	1.24 (1.07–1.41)	0.29	1.01 (0.89–1.26)	0.84 (0.60–1.08)	0.31
CD4 (10^3^/µl)	0.87 (0.82–0.92)	0.77 (0.64–0.91)	0.18	0.86 (0.81–0.90)	0.79 (0.68–0.89)	0.22	0.64 (0.55–0.72)	0.51 (0.33–0.68)	0.26
CD8 (10^3^/µl)	0.47 (0.44–0.51)	0.47 (0.37–0.57)	0.91	0.47 (0.44–0.51)	0.48 (0.40–0.57)	0.87	0.37 (0.32–0.42)	0.38 (0.22–0.55)	0.81
CD19 (10^3^/µl)	0.18 (0.16–0.20)	0.14 (0.15–0.18)	0.08	0.17 (0.15–0.18)	0.13 (0.09–0.16)	0.09	**0.12 (0.10–0.14)**	**0.08 (0.06–0.09)**	** < 0.001**
CD4/CD8 ratio	**1.98 (1.84–2.12)**	**1.71 (1.53–1.90)**	**0.023**	**1.95 (1.82–2.08)**	**1.70 (1.51–1.90)**	**0.032**	1.93 (1.75–2.11)	1.51 (1.10–1.91	0.09
NK (10^3^/µl)	0.16 (0.14–0.18)	0.12 (0.85–0.16)	0.11	0.13 (0.12–0.14)	0.11 (0.08–0.14)	0.14	0.14 (0.12–0.16)	0.14 (0.07–0.21)	0.99
LUC (%)	1.31 (1.19–1.44)	1.13 (0.86–1.40	0.25	1.55 (1.40–1.70)	1.74 (1.37–2.11)	0.34	1.22 (1.07–1.37)	1.04 (0.79–1.30)	0.32
IL-6 (pg/mL)	45.50 (21.40–69.60)	29.17 (3.66–54.68)	0.56	62.81 (16.45–109.16)	44.14 (2.72–85.56)	0.72	114.39 (52.90–175.88)	61.43 (16.66–106.19)	0.46
IL-10 (pg/mL)	71.47 (13.72–129.22)	52.39 (− 1.78–106.57)	0.78	58.06 (29.30–86.32)	222.52 (− 77.39–522.44)	0.24	161.57 (78.57–244.58)	116.71 (− 40.49–223.92)	0.63
TNF (pg/mL)	13.44 (7.86–19.02)	23.54 (2.44–44.63)	0.17	18.36 (12.29–24.43)	18.93 (− 50.27–88.12)	0.96	16.66 (9.16–24.16)	21.25 (2.83–39.67)	0.58

Results are expressed as mean (95% confidence interval). Bold: P-value < 0.05. *NK* natural killer cells, *LUC* large unstained cells, *IL* interleukin

About the effect of *PPARG* rs1801282 genotypes on levels of immune cells [including total leukocytes, granulocytes (neutrophils, eosinophils and basophils), agranulocytes (monocytes, lymphocytes) and LUC%], in general there was no effect on those levels by *PPARG* Pro12Ala SNP distribution (p > 0.05), except at 34 weeks of gestation where statistically significant differences were found in leukocytes and neutrophils levels, being lower in carriers of Pro12Ala genotype (p = 0.028 and p = 0.031, respectively).

On the other hand, statistically significant differences were revealed in B-lymphocyte (CD19) counts and CD4/CD8 ratio between mothers carrying the *CC* and the *CG* genotypes: results show significant changes in CD19 cells at delivery (p < 0.001) and marginally significant at 24 and 34 weeks of pregnancy. Conversely, CD4/CD8 ratio varied significantly at 24 and 34 weeks of pregnancy (p = 0.012 and p = 0.030, respectively) between mothers with different genotypes, and also at delivery, although not reaching statistical significance in this last case. In these weeks of pregnancy (24 and 34) and at delivery, CD19 levels and CD4/CD8 ratio were increased in homozygous mothers compared against heterozygous mothers (Table 2). Finally, levels of cytokines such as TNF, IL-6 and IL-10 seem to be increased by the end of pregnancy; although no statistically significant effect of *PPARG* rs1801282 genotypes on levels of those cytokines were observed in our cohort (Table 2). Other immune molecules such as CD3, CD4 and CD8 were not found to be influenced by the genotypes in mothers during pregnancy and at delivery in our study.

Regarding babies born to mothers participating in the study, genotype frequencies were: 80 (89.89%) *CC*, 8 (8.99%) *CG* and 1 (1.12%) *GG*; allele frequencies were: 93.85% *C* and 6.15% *G*, and no deviation from Hardy–Weinberg equilibrium was detected (p = 0.15). Due to low frequency of minor homozygotes in the newborns, subsequent statistical analyses were done comparing *CG* + *GG vs. CC*. No statistically significant variation (p > 0.05) has been found in cord blood levels of immune cells or cytokines between babies who carry *CC* major homozygous genotype and carriers of *G* (*CG* + *GG*) (Table 3). Levels of some granulocytes (eosinophils and basophils) and agranulocytes (monocytes, lymphocytes), as well as the lymphocyte subsets CD3, CD4, CD8 and CD19 and CD4/CD8 ratio, are slightly increased compared to mothers at week 40 of pregnancy. On the contrary, neutrophils and LUC% decreased compared to mothers at time of delivery. Nevertheless, results obtained for newborn cord blood are in normal range according to clinical reference values.

**Table 3:** Immunological parameters in newborn cord blood according to rs1801282 PPARG genotype

Immunological parameters	Pro12Pro (*CC*) n = 79	Pro12Ala (*CG* + *GG*) n = 9	*p*
Leukocytes (10^3^/µl)	13.84 (12.72–14.96)	13.95 (9.30–18.60)	0.95
Neutrophils (10^3^/µl)	7.41 (6.57–8.26)	6.94 (3.08–10.80)	0.73
Monocytes (10^3^/µl)	1.06 (0.94–1.18)	1.05 (0.64–1.45)	0.94
Eosinophils (10^3^/µl)	0.47 (0.41–0.53)	0.48 (0.16–0.80)	0.90
Basophils (10^3^/µl)	0.20 (0.17–0.24)	0.21 (0.11–0.31)	0.92
Lymphocytes (10^3^/µl)	4.40 (3.99–4.82)	5.07 (3.56–6.58)	0.32
CD3 (10^3^/µl)	2.46 (2.21–2.71)	2.89 (1.79–3.98)	0.27
CD4 (10^3^/µl)	1.65 (1.47–1.83)	2.11 (1.32–2.90)	0.11
CD8 (10^3^/µl)	0.66 (0.55–0.77)	0.67 (0.35–0.98)	0.95
CD19 (10^3^/µl)	0.57 (0.45–0.68)	0.62 (0.26–0.97)	0.77
CD4/CD8	2.86 (2.57–3.16)	3.44 (2.31–4.57)	0.21
NK (10^3^/µl)	0.88 (0.71–1.06)	1.17 (0.58–1.76)	0.29
LUC (%)	0.31 (0.26–0.36)	0.27 (0.14–0.39)	0.60

Results are expressed as mean (95% confidence interval). *NK* natural killer cells, *LUC* large unstained cells.

## Discussion

The main goal of the current study was to determine if rs1808212 *PPARG* polymorphism could be associated with immune cell and cytokine levels in mothers during gestation and in newborn cord blood. Our results suggest that during pregnancy, the majority of immune components are not altered in Ala12 *PPARG* carrier mothers with respect to Pro12Pro subjects. However, CD19 molecules seem to vary slightly at second and third trimester of pregnancy and at time of delivery, although not reaching statistically significant differences. B-lymphocyte antigen CD19 is a clinical biomarker linked to the lymphocyte B development, with relevance during early life. CD19 is a transmembrane protein which acts as an adaptor protein to recruit cytoplasmic signaling proteins to the membrane. Moreover, CD19 is critically involved in establishing intrinsic B cell signaling thresholds through modulating both B cell receptor dependent and independent signaling. It plays roles in the antigen-independent development, as well as the immunoglobulin-induced activation of B cells. CD19 is thus critical for the body to mount an optimal immune response [32].

Results also suggest that CD4/CD8 ratio may be influenced by *PPARG* Pro12Ala polymorphism during pregnancy, as there are statistically significant differences in mothers’ carriers of *CC vs*. carriers of *CG* genotypes in weeks 24 and 34, although not at 40 weeks. CD4 helper/inducer cells and CD8 cytotoxic/suppressor cells are T lymphocytes phenotypes, characterized by distinct surface markers and functions that mostly reside in lymph nodes but also circulate in the blood. CD4/CD8 ratios between 1.5 and 2.5 are generally considered normal; however, a wide heterogeneity exists because sex, age, ethnicity, genetics, exposures, and infections may all impact the ratio. A low or inverted CD4/CD8 ratio is an immune risk phenotype and is associated with altered immune function, immune senescence, and chronic inflammation [33]. Thus, the mothers with the non-prevalent genotype present a borderline normal ratio. Nonetheless, despite statistically significant results obtained in the current study, values obtained in the *CC* and *GC* genotypes at the different times of the study overlap, so the differences may not be biologically significant.

Nuclear receptor PPARG has a major role in transcriptional regulation of genes involved in various physiologic processes of clinical significance, such as developmental, metabolic and anti-inflammatory processes. PPARG plays a pivotal role in adipogenesis, fatty acid uptake, lipid storage and systemic energy homeostasis, as well as in the control of inflammation [34]. *PPARG* is expressed in blood cells and induced during macrophage differentiation [35]. It enhances macrophage lipid uptake and has anti-inflammatory effects [36], which are closely linked with its anti-diabetic effects [37]. *PPARG* is expressed in B lymphocytes [38] and T lymphocytes [39].

PPARG has anti-inflammatory properties and its ligands have been demonstrated to suppress production of monocyte/macrophages inflammatory cytokines such as TNF, IL-6, and IL-1β through inhibiting the activity of transcription factors such as nuclear factor κB, activator protein-1, and signal transducers and activators of transcription [10, 40, 41]. No statistically significant differences in levels of TNF, IL-6, and IL-10 cytokines in mother carriers of *CG* compared to those carrying *CC* genotype have been found at any time of pregnancy studied.

*PPARG* Ala12 allele has been related to increased long-term weight gain [42], and also with a reduction in type 2 diabetes risk [30]. In our study, mothers who carried CG genotype were less prone to the development of diabetes during pregnancy against those who carried CC (26.32% *vs.* 30.51%), although not reaching statistical significance, it has been reported that subjects carrying Pro allele have increased diabetes risk [40, 43–45]. Other study reported that gestational diabetes mellitus is associated with increased leukocyte PPARG expression (46), which could explain the slight effects of *PPARG* polymorphism on lymphocytes subsets observed in our study. In this regard, as subject number involved in the current study is limited, replication in a larger population is needed since reduced transcriptional activity of the *PPARG* gene result of Pro to Ala aminoacid substitution has been demonstrated [5]. Results from a large scale meta-analysis shown that *PPARG* (rs1801282) was significantly associated with increased/decreased risk of GDM in Asian population, mostly from China, but not in Caucasian population [47].

Birth weight is slightly higher in the offspring of heterozygous mothers compared to homozygous pregnant women. However, no differences in newborn weight were found when comparing newborns *CC* carriers *vs*. *CG* + *GG*, as described above. These results are in line with a study in a large German cohort including mother/child pairs showing that *PPARG* Pro12Ala polymorphism is not relevant for intrauterine growth, although effects developed later in life depending on predisposition or environmental factors could not be discarded [48]. However, it is opposed to another study in 208 Finnish subjects in which they conclude that Ala12 allele of *PPARG* gene is associated with high weight and ponderal index at birth [49]. Since PPARG is involved in glucose and lipid metabolism, it could be considered as an important molecular factor participating in these metabolic pathways during pregnancy. Indeed, decreased *PPARG* mRNA and protein levels have been observed in subcutaneous adipose tissue from obese gestational diabetic women, suggesting that this change might be part of the molecular mechanism to accelerate fat catabolism and thereby ensure fetal nutrition in late gestation [50].

Some limitations in the present study should be noted, as the sample size is relatively low, although sample characteristics are quite homogeneous: all the mother–child pairs where from Spanish origin and no statistically significant differences were found among groups depending on rs1808212 genotype distribution in clinical or demographic characteristics. No data is available about PPARG expression in our study population. Moreover, blood counts and cytokine levels data were not available at the beginning of the pregnancy. Thus, the study results should be taken with caution, and should be replicated in a larger population. Given the pivotal role that PPARG plays in lipid storage, systemic energy homeostasis and in the control of inflammation as well, more research is warranted in this field.

## Conclusions

In summary, results suggest that *PPARG* Pro12Ala polymorphism might be one of the factors that would determine higher CD19 cells during pregnancy in homozygous *PPARG* rs1808212 SNP mothers, especially at delivery, compared to heterozygous participants. Additionally, CD4/CD8 ratio was also slightly higher in mothers with Pro12Pro genotype at second and third trimester of pregnancy, but not at the time of delivery (40 weeks). Besides, our investigation reveals that no other immune molecular and cellular components seems to be altered by the Ala allele of rs1808212 *PPARG* polymorphism in mothers at 24 and 34 weeks of pregnancy. However, as newborn innate immune system does not seems to be influenced by *PPARG* Pro12Ala polymorphism in cord blood, more research is warranted to clarify the role of *PPARG* on immune parameters and their relationship with inflammatory response during pregnancy.

## Data Availability

The datasets generated and/or analysed during the current study are not publicly available due to nature of the information that could compromise research participant privacy/consent, but are available from the corresponding author on reasonable request.
